# High resolution HLA-DRB1 analysis and shared molecular amino acid signature of DRβ1 molecules in Occult hepatitis B infection

**DOI:** 10.1186/s12865-022-00496-2

**Published:** 2022-04-25

**Authors:** Tianju Wang, Chunmei Shen, Hengxin Li, Liping Chen, Sheng Liu, Jun Qi

**Affiliations:** 1 HLA Typing Laboratory, Blood Center of the Shaanxi Province, Institute of Xi’an Blood Bank, 407# Zhuque Ave, Xi’an, Shaanxi Province 710061 People’s Republic of China; 2 Key Laboratory of Environment and Gene Related to Diseases, Xi’an Jiaotong University, Xi’an, Shaanxi 710061 People’s Republic of China

**Keywords:** Occult hepatitis B infection, Human leukocyte antigen, Amino acid variation, Peptide binding pockets

## Abstract

**Aim:**

To investigate the association of human leukocyte antigen (HLA)-DRB1 alleles and the variations of polymorphic amino acid changes in DRβ1 chain in Shaanxi Han population with Occult hepatitis B infection (OBI).

**Methods:**

High-resolution HLA-DRB1 genotyping was performed in 107 OBI carriers and 280 normal controls. Sequence information was used to assign which amino acids were encoded at all polymorphic positions. Three-dimensional modeling was performed to explore the effect of the key residues on the HLA-DRB1 molecule.

**Results:**

Strong susceptible association for allele DRB1*07:01 was observed in OBI carriers. The amino acid variation at HLA-DRβ1 molecule revealed susceptible associations for residues Gln^4β^, Val^57β^(P9), Ser^60β^(P9) and Val^78β^(P4), the amino acids Arg^4β^, Asp^57β^(P9), Tyr^60β^(P9) and Tyr^78β^(P4) showed protective associations.

**Conclusion:**

Alleles DRB1*07:01 showed strong susceptible associations in OBI carriers. The amino acid variations in DRβ molecules revealed significant molecular markers for susceptibility and protection from OBI in Shaanxi Han population.

## Introduction

Occult hepatitis B virus infection (OBI) is a special type of HBV infection. When the infection is excluded during the window period, the HBsAg is negative in the serum, while HBV DNA is positive (the concentration of HBV DNA in the serum, or liver tissue, lymphatic system cells < 200 IU/ml) [1, 2]. The prevalence of OBI among blood donors in different parts is quite variable depending on the HBV endemicity. The OBI prevalence rates of 1:7517, 1:8209 and 1:9819 were observed among blood donors in China, South African and Europeans [3–5]. The prevalence of OBI is higher in chronic liver disease and may be as high as 40 to 75% in HBsAg-negative hepatocellular carcinoma [6]. OBI may impact in several different clinical contexts, including the possible transmission of the infection [7, 8], the risk of reactivation [9], the contribution to liver disease progression and to the development of hepatocellular carcinoma [10].

The mechanisms of OBI have not been completely understood. Studies have showed that HBV replication and transcription have been strongly suppressed by the host immune response which consists mainly of anti-viral cytotoxic T cells [11, 12]. Furthermore, OBI carriers with anti-HBc show a typical T cells memory response [13].

Human leukocyte antigen (HLA) plays an important role in anti-viral immunity [14]. HLA class II molecules recognize and bind to viral antigen peptides, then present them to CD4 + T cells. As a result, B cells produce anti-viral antibodies. In addition, the HLA-DRB1 molecule is a heterodimer, composed of an α and a β chain; the heterodimer forms a peptide-binding groove consisting of nine peptide residue positions (P1-P9), of which positions P1, P4, P6, P7 and P9 constitute peptide anchoring pockets, for the various candidate antigenic peptides. The peptide-binding groove of HLA-DRB1 molecular accommodates 13–18 amino acid peptides. HLA molecular differing only at 1–2 amino acids showed different peptide binding preference. Lacking of evaluating the relationship of HLA-DRB1 alleles with OBI in Chinese Han population. The association between HLA-DRB1 amino acid signatures of the peptide-binding pockets and OBI infections has not been characterized previously.

In this study, we analyzed the risk HLA-DRB1 alleles in a case–control study in the Shaanxi Han populations associated with OBI. We also reported differences in the amino acid signatures of peptide-binding pockets of the HLA-DRB1 molecules in OBI carriers as compared with controls. Determination of the structural of DRB1 molecules associated with OBI help in identifying the disease mechanism and deepening understanding of OBI etiology.

## Materials and methods

### Subjects

178,941 healthy blood donors were collected from the Shaanxi Blood Center during the period from 1 January 2013 to 31 December 2013. All blood donors have no underlying diseases. Samples that had confirmatory HBsAg (−)/HBV DNA (+) were detected anti-HBs, HBeAg, anti-HBe and anti-HBc. Samples with HBsAg (−)/HBV DNA (+) and serologic markers (+) were collected. Samples with HBsAg (−)/HBV DNA (+) and serologic markers (−) were tested again after six months and only markers that were negative were collected.

A total of 107 blood samples which showed negative for anti-hepatitis C virus (HCV), anti-human immunodeficiency virus (HIV) and HBsAg while positive for HBV NDA were collected from the OBI infection volunteers during January to December in 2013. 280 healthy control subjects were involved from voluntary blood donors whose bloods were negative for anti-HCV and anti-HIV, HBsAg and HBV DNA.

### HLA-DRB1 data

High-resolution HLA-DRB1 genotypes were available from a previous study [15]. High-resolution DRB1 sequencing was completed for all study participants using an AlleleAEQR HLA-DRB1 reagent kit and protocol (One Lambda, INC., Canoga Park, CA, USA) and an ABI 3730xl automated sequencer (Applied Biosystems, Foster City, CA). Four-digit DRB1 genotypes were assigned using Assign software (ThermoFisher, Waltham, USA).

### Sequence’s alignment and 3D protein structure modeling of HLA-DRB1 molecules

Sequences of DRB1 alleles were obtained from the IMGT/HLA database release 3.39.0, January 2020 release (http://raw.githubusercontent.com/ANHIG/IMGTHLA/Latest/alignments/DRB1_pro.txt). Each individual was assigned two amino acids for each polymorphic residue. HLA-DRB1 proteins of known structure suitable as modeling templates were identified in the Protein Data Bank (PDB; http://www.rcsb.org/pdb/) and evaluated for structural quality. The binding pockets were identified based on the annotation from Stern et al. [16]. The structure model of OBI-susceptible HLA-DRB1 molecules were constructed based on the x-ray diffraction structure of HLA-DR1 in complex with an endogenous peptide [PDB ID: 1AQD].

### Statistical analysis

The differences in allele frequencies of HLA-DRB1 between OBI carriers and controls were compared by chi-squared statistics. Odds ratio (OR) and 95% confidence intervals (95%CI) were calculated for each allele. All amino acid polymorphic positions derived from exon 2 of HLA-DRB1 were tested for their involvement as risk factors for the development of OBI. The association of amino acid residue carrier frequencies were analyzed using Fisher’ s exact test with 2 × 2 contingency table. Adjustment for multiple comparisons were conducted with Bonferroni method, corrected *P* (*Pc*) values were calculated by multiplying the *P* value by the number of tested amino acid positions [17]. All the statistical analyses were carried out using the SPSS (SPSS Inc, Chicago, IL) and Graphad Prism (Graphad software Inc, San Diego, CA) packages.

## Results

### Clinical characteristics of subjects

A total of 387 blood donor (107 OBI carriers and 280 normal controls) were enrolled in this study. General information about the subject was provided in Table 1. The sex, age, address and type of donor distributions showed no statistical difference between the OBI carriers and normal controls. All participants were Chinese Han living in the northwest of China. Among 107 cases of OBI, 104 (97.20%) were positive for either anti-HBs, anti-HBc, anti-HBe, or both. 48 (44.86%) of the OBI cases were positive for both anti-HBs and anti- HBc, while 6 (5.61%) were positive for only anti-HBs, and 37 (34.58%) were positive for only anti-HBc. 3 (2.80%) of the 107 cases of OBI were negative for all serological markers.

**Table 1:** The characteristics of the subjects

General information	OBI group (n = 107) (%)	Control group (n = 280) (%)	*P*
*Age*			
≤ 40	62 (57.94)	185 (66.07)	0.137
> 40	45 (42.06)	95 (33.93)	
*Sex*			
Male	75 (70.09)	174 (62.14)	0.144
Female	32 (29.91)	106 (37.86)	
*Address*			
City	59 (55.14)	150 (53.57)	0.782
Country	48 (44.86)	130 (46.43)	
*Type of donor*			
First-time donor	82 (76.64)	209 (74.64)	0.685
Repeat donor	25 (23.36)	71 (25.36)	
*Serological markers*			
Anti-HBs (+)	6 (5.61%)	–	
Anti-HBs (+) anti-HBe (+) anti-HBc (+)	6 (5.61%)	–	
Anti-HBe (+) anti-HBc (+)	1 (0.93%)	–	
Anti-HBs (+) anti-HBc (+)	48 (44.86%)	–	
Anti-HBc (+)	37 (34.58%)	–	
Anti-HBe (+)	2 (1.87%)	–	
Anti-HBs (+) anti-HBe (+)	4 (3.74%)	–	

Comparison of the frequency of HLA-DRB1 alleles in OBI carriers and control group at high resolution

**Fig. 1:**
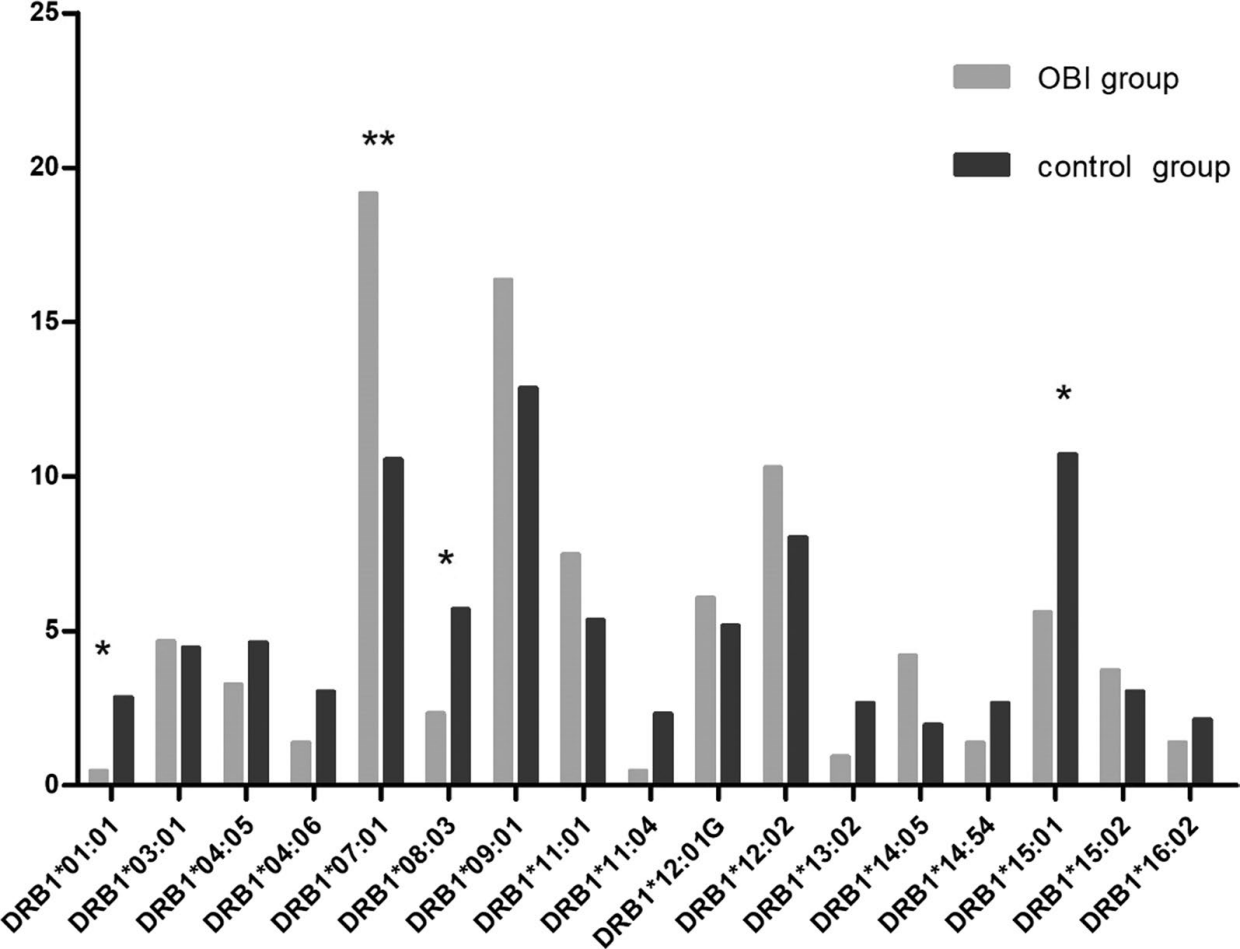
Comparisons of the frequencies of HLA-DRB1 alleles between OBI carriers and controls. Frequencies of HLA-DRB1 alleles between OBI carriers (gray column) and control subjects (black column) were compared. Alleles with > 2% frequencies in either group were shown. *P* values for multiple comparisons (*Pc*) were corrected by Bonferroni correction. *P < 0.05, **Pc < 0.05

As shown in Table 2 and Fig. 1, forty-two HLA-DRB1 alleles were detected, but only 16 had a frequency > 2%. Among of 16 DRB1 alleles, four showed a significantly different distribution between OBI carriers and controls, including HLA-DRB1*07:01, HLA-DRB1*01:01, HLA-DRB1*08:03, HLA-DRB1*15:01.

**Table 2:** Comparison of the frequency of HLA-DRB1 alleles in OBI carriers and control group at high resolution

	OBI (GF%)	Control (GF%)	*P* value	*Pc* value	OR	95% CI
HLA	2n = 214	2n = 560				
DRB1*01:01	0.47	2.86	0.042	1.764	0.160	0.021–1.211
DRB1*01:02	0.47	0.36	–	–	–	–
DRB1*03:01	4.67	4.46	0.901	–	–	–
DRB1*04:01	0.00	1.43	–	–	–	–
DRB1*04:03	0.93	1.43	–	–	–	–
DRB1*04:04	0.47	0.71	–	–	–	–
DRB1*04:05	3.27	4.64	0.398	–	–	–
DRB1*04:06	1.40	3.04	0.200	–	–	–
DRB1*04:07	0.47	0.18	–	–	–	–
DRB1*04:08	0.93	0.18	–	–	–	–
DRB1*04:10	0.00	0.71	–	–	–	–
DRB1*07:01	**19.16**	**10.54**	**0.001**	**0.042**	**2.012**	**1.303–3.107**
DRB1*08:01	0.47	0.00	–	–	–	–
DRB1*08:02	0.47	1.07	–	–	–	–
DRB1*08:03	2.34	5.71	0.049	2.058	0.395	0.152–1.027
DRB1*08:09	0.47	0.18	–	–	–	–
DRB1*09:01	16.36	12.86	0.207	–	–	–
DRB1*10:01	0.47	1.79	–	–		
DRB1*11:01	7.48	5.36	0.265	–	–	–
DRB1*11:03	0.00	0.18	–	–	–	–
DRB1*11:04	0.47	2.32	0.128	–	–	–
DRB1*12:01G	6.07	5.18	0.623	–	–	–
DRB1*12:02	10.28	8.04	0.321	–	–	–
DRB1*13:01	0.93	1.61	–	–	–	–
DRB1*13:02	0.93	2.68	0.176	–	–	–
DRB1*13:05	0.47	0.00	–	–	–	–
DRB1*13:07	0.00	0.18	–	–	–	–
DRB1*13:12	0.47	0.18	–	–	–	–
DRB1*13:49	0.47	0.00	–	–	–	–
DRB1*14:03	0.93	0.18	–	–	–	–
DRB1*14:04	0.00	0.71	–	–	–	–
DRB1*14:05	4.21	1.96	0.079	–	–	–
DRB1*14:07	0.00	0.18	–	–	–	–
DRB1*14:09	0.47	0.00	–	–	–	–
DRB1*14:11	0.00	0.18	–	–	–	–
DRB1*14:12	0.00	0.18	–	–	–	–
DRB1*14:25	0.00	0.18	–	–	–	–
DRB1*14:54	1.40	2.68	0.292	–	–	–
DRB1*15:01	5.61	10.71	0.029	1.218	0.495	0.261–0.940
DRB1*15:02	3.74	3.04	0.621	–	–	–
DRB1*15:04	1.87	0.00	–	–	–	–
DRB1*16:02	1.40	2.14	0.504	–	–	–

OBI, occult hepatitis B infection; GF, gene frequency; OR, odds ratio; 95%CI, 95% confidence interval; *Pc*, corrected *P*

Only alleles with > 2% frequencies in either group were shown analyzed. – indicated the allele frequency < 2% were not analyzed, or *P* > 0.05, *Pc* were not calculated. In bold: significant value of *Pc* < 0.05

The frequency of the allele HLA-DRB1*07:01 (19.16% vs 10.54%, *P* = 0.001, OR = 2.012, 95% CI 1.303–3.107) was higher in OBI carriers than in healthy controls. After Bonferroni correction, the associations remained significant for the allele HLA-DRB1*07:01 (*Pc* = 0.042) that was related to susceptibility to OBI.

In contrast, the alleles HLA-DRB1*01:01 (0.47% vs 2.86%, *P* = 0.042, OR = 0.160, 95% CI 0.021–1.211), HLA-DRB1*08:03(2.34% vs 5.71%, *P* = 0.049, OR = 0.395, 95% CI 0.152–1.027), HLA-DRB1*15:01(5.61% vs10.71%, *P* = 0.029, OR = 0.495, 95% CI 0.261–0.940) were more frequent in the control group. After Bonferroni correction, there was no allele association with OBI.

### Homozygous and heterozygous of HLA-DRB1*07:01 and DRB1*12:02 in OBI carriers and controls

The analysis for homozygous and heterozygous alleles combination of HLA-DRB1*07:01 and DRB1*12:02 is presented (Table 3). The frequency of HLA-DRB1*07:01^+^/07:01^+^ (4.67% vs 0.71%, *P* = 0.192, OR = 6,814, 95% CI 1.301–35.690) was higher in OBI carriers than in healthy controls. In contrast, HLA-DRB1*07:01^−^/07:01^−^ (66.36% vs 79.39%, *P* = 0.012, OR = 0.515, 95% CI 0.314–0.845) was more frequent in the control group. The differences in frequencies of DRB1*12:02^+^/DRB1*12:02^+^, DRB1*12:02^+^/DRB1*12:02^−^ and DRB1*12:02^−^/DRB1*12:02^−^ were not significant.

**Table 3:** Homozygous and heterozygous of HLA-DRB1*07:01 and DRB1*12:02 in OBI carriers and controls

HLA genotypes	OBI (GF%) n = 107	Control (GF%) n = 280	*P*	OR	95%CI
DRB1*07:01^+^/DRB1*07:01^+^	5 (4.67)	2 (0.71)	0.019	6.814	1.301–35.690
DRB1*07:01^+^/DRB1*07:01^−^	31 (28.97)	56 (20.00)	0.076	1.632	0.979–2.718
DRB1*07:01^−^/DRB1*07:01^−^	71 (66.36)	222 (79.39)	0.012	0.515	0.314–0.845
DRB1*12:02^+^/DRB1*12:02^+^	0	0	–	–	–
DRB1*12:02^+^/DRB1*12:02^−^	22 (20.56)	44 (15.71)	0.290	1.388	0.786–2.452
DRB1*12:02^−^/DRB1*12:02^−^	85 (79.44)	236 (84.29)	0.290	0.720	0.408–1.272

OBI, occult hepatitis B infection; GF, gene frequency; OR, odds ratio; 95%CI, 95% confidence interval.

In bold: significant value of *Pc* < 0.05

### Susceptible of HLA-DRB1 amino acid residues associations with OBI

**Fig. 2:**
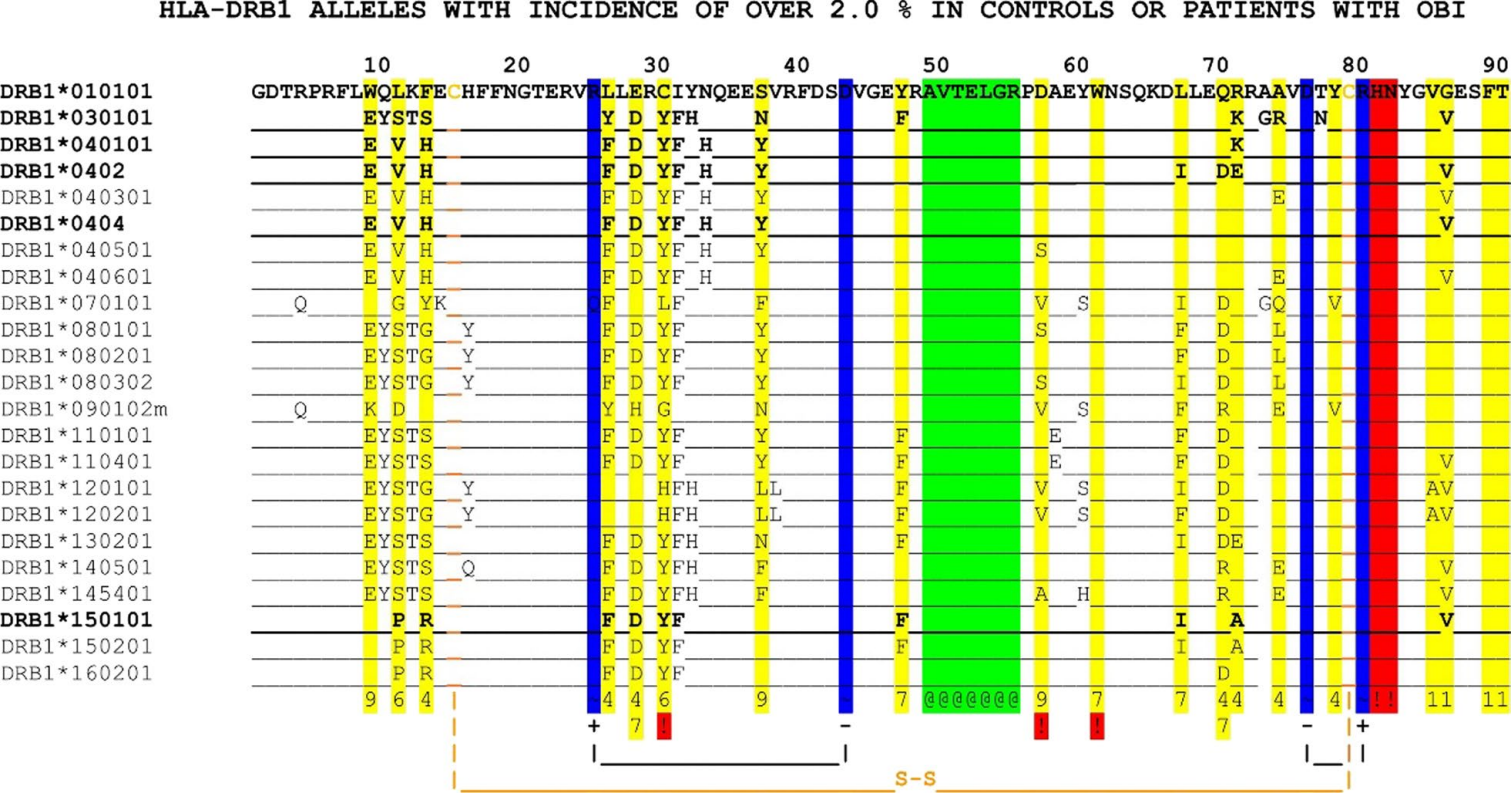
HLA-DRB1 alleles with incidence of over 2% in controls or patients with OBI 1. Alleles with known crystal structure are in bold. 2. Identity in residues is indicated by _, while unknown residues are shown as *. 3. Symbols below the sequence of the last allele: For antigen binding (highlighted in yellow): residues participating in the formation of a particular pocket indicated by the number of the pocket (1, 4, 6, 7, 9) at the bottom of the column; in case a residue participates in more than one pockets this is indicated by numbers on two lines. For interchain interactions:!, residues forming hydrogen bonds with antigenic peptide backbone, or interchain salt bridges via oppositely charged side chains (highlighted in red). Disulfide bridges in orange. ~ : intra-chain salt bridges marked with respective charge signs are in blue. G^11β^, Y^13β^, K^14β^, Q^25β^, L^30β^, Q^74β^ have not found in any of the other alleles. Modified from http://pubmed.ncbi.nlm.nih.gov/17497145/

Unequivocal sequences were obtained in all 107 OBI carriers and in 280 controls. Sequencing analysis of the polymorphic exon 2 of the DRB1 gene showed 31 polymorphic amino acid positions. HLA-DRB1 alleles with incidence of over 2% in controls or patients with OBI were shown in Fig. 2.

Significant differences in the frequencies of amino acid between OBI carriers and controls were observed at 13 of the 31 polymorphic amino acids, at positions 4, 11, 13, 14, 25, 28, 30, 37, 57, 60, 73, 74 and 78. The results of amino acid variation for each position are provided in Table 4. After Bonferroni correction: Gln-4(*P* = 0.0010, *Pc* = 0.031, OR = 1.804, 95% CI 1.282–2.538), Val-57(*P* = 0.0002, *Pc* = 0.062, OR = 1.866, 95% CI 1.357–2.566), Ser-60 (*P* = 0.0002, *Pc* = 0.062, OR = 1.866, 95% CI 1.357–2.566), Val-78(*P* = 0.0010, *Pc* = 0.031, OR = 1.804, 95% CI 1.282–2.538), four amino acid positions showed the strongest susceptible associations with OBI carriers.

**Table 4:** Susceptible of HLA-DRB1 amino acid residues associations with OBI

Amino acid position	Amino acid variants	OBI (AA-F%) 2n = 214	Control (AA-F%) 2n = 560	*p* value	*Pc*	OR	95% CI
**4**	**Glutamine (Q)**	**76 (35.51)**	**131 (23.39)**	**0.0010**	**0.031**	**1.804**	**1.282–2.538**
	**All others**	**138 (64.49)**	**429 (76.61)**				
11	Glycine (G)	41 (19.16)	59 (10.54)	0.0025	0.0775	2.012	1.303–3.108
	All others	173 (80.84)	501 (89.46)				
13	Tyrosine (Y)	41 (19.16)	59 (10.54)	0.0025	0.0775	2.012	1.303–3.108
	All others	173 (80.84)	501 (89.46)				
14	Lysine (K)	41 (19.16)	59 (10.54)	0.0025	0.0775	2.012	1.303–3.108
	All others	173 (80.84)	501 (89.46)				
25	Glutamine (Q)	41 (19.16)	59 (10.54)	0.0025	0.0775	2.012	1.303–3.108
	All others	173 (80.84)	501 (89.46)				
28	Glutamic acid (E)	81 (37.85)	163 (29.11)	0.0244	0.7564	1.483	1.065–2.065
	All others	133 (62.15)	397 (70.89)				
30	Leucine (L)	41 (19.16)	59 (10.54)	0.0025	0.0775	2.012	1.303–3.108
	All others	173 (80.84)	501 (89.46)				
37	Phenylalanine (F)	54 (25.23)	92 (16.43)	0.0074	0.2294	1.717	1.173–2.513
	All others	160 (74.77)	468 (83.57)				
**57**	**Valine (V)**	**111 (51.87)**	**205 (36.61)**	**0.0002**	**0.0062**	**1.866**	**1.357–2.566**
	**All others**	**103 (48.13)**	**355 (63.39)**				
**60**	**Serine (S)**	**111 (51.87)**	**205 (36.61)**	**0.0002**	**0.0062**	**1.866**	**1.357–2.566**
	**All others**	**103 (48.13)**	**355 (63.39)**				
73	Glycine (G)	51 (23.83)	84 (15.00)	0.0058	0.1798	1.762	1.192–2.604
	All others	164 (76.64)	476 (85.00)				
74	Glutamine (Q)	41 (19.16)	59 (10.54)	0.0025	0.0775	2.012	1.303–3.108
	All others	173 (80.84)	501 (89.46)				
**78**	**Valine (V)**	**76 (35.51)**	**131 (23.39)**	**0.0010**	**0.031**	**1.804**	**1.282–2.538**
	**All others**	**138 (64.49)**	**429 (76.61)**				

OBI, occult hepatitis B infection; AA-F, amino acid frequency; OR, odds ratio; 95%CI, 95% confidence interval; *Pc*, corrected *P*.

In bold: significant value of *Pc* < 0.05

### Specific amino acid residues of HLA-DRB1 mediate risk and protection in OBI

**Fig. 3:**
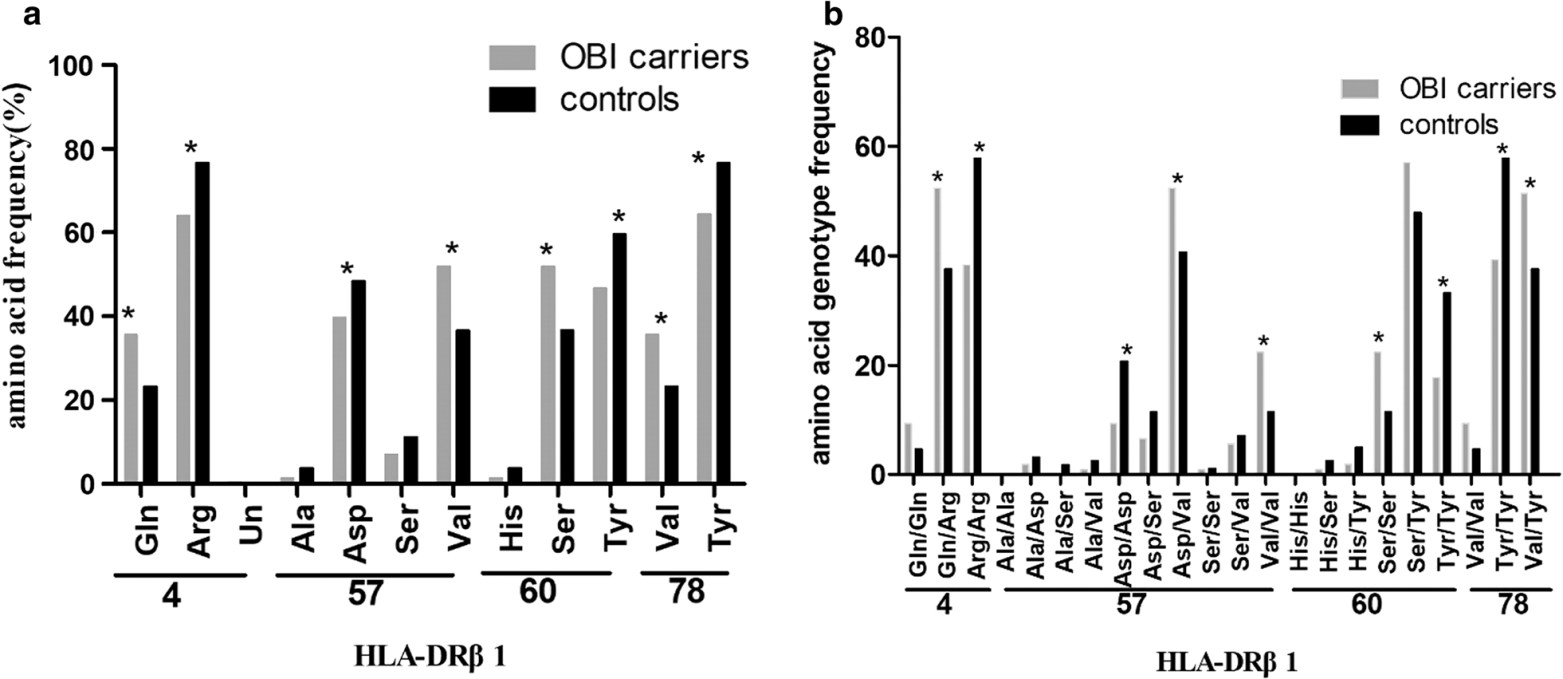
**a** Amino acid frequencies of HLA-DRB1 at position 4, 57, 60, 78 between OBI carriers (gray column) and controls (black column). **b** Amino acid genotype frequencies of HLA-DRB1 at position 4, 57, 60, 78 between OBI carriers (gray column) and controls (black column). Gln: glutamine; Arg: Arginine; Un: Undetermined; Ala: alanine; Ser: Serine; Val: Valine; His: Histidine; Tyr: Tyrosine. *P < 0.05

**Fig. 4:**
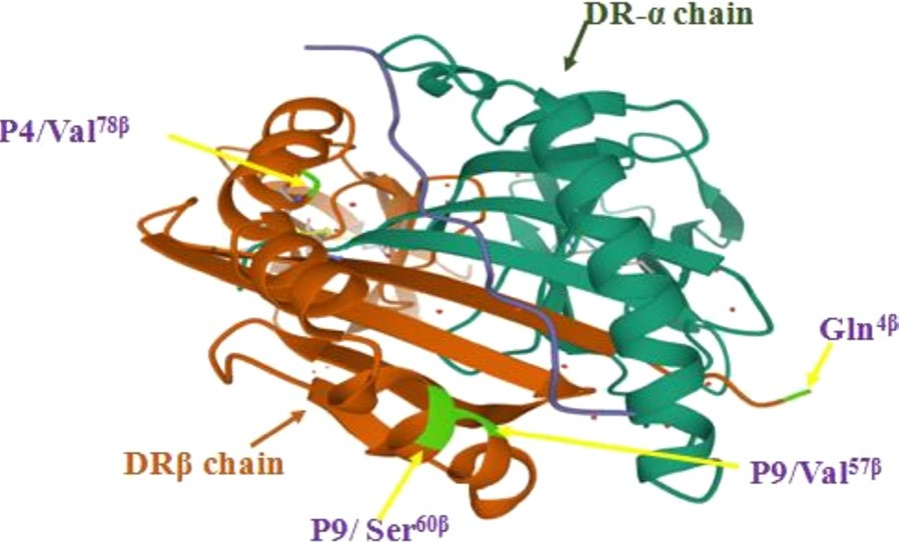
Three-dimensional ribbon models of the HLA proteins associated with OBI. The protein structures of HLA-DRB1 are based on Protein Data Bank (PDB) entries 1AQD. Antigen peptides are shown in blue which was previously shown to bind to HLA-DRB1*01:01. DR α chain is showed by green cyan, while β chain is showed by orange. The four critical residues Gln4β, Val57β(P9), Ser60β(P9) and Val78β(P4) are shown by yellow arrows. The residues in the binding groove of the HLA were selected by proximity (2.45 Å) to the peptide

Four amino acids at the same four positions showed the strongest protective effects (Table 5, Figs. 3a, 4): Arg at position 4 (*P* = 0.0006), Asp at position 57 (*P* = 0.0359), Tyr at position 60 (*P* = 0.0016), Tyr at position 78 (*P* = 0.0010). Other significant amino acids that conferred either susceptibility to or protection from OBI carriers were shown in Tables 4 and 5.

**Table 5:** HLA-DRB1 amino acid variants that showed strong association with OBI carriers

Amino acid position	Amino acid variants	OBI (AA-F%) 2n = 214	Control (AA-F%) 2n = 560	*p* value	OR	95% CI
4	**Glutamine (Q)**	**76 (35.51)**	**131 (23.39)**	**0.0010**	**1.804**	**1.282–2.538**
	**Arginine (R)**	**137 (64.02)**	**429 (76.61)**	**0.0006**	**0.5433**	**0.3863–0.7641**
	Undetermined	1 (0.47)	0 (0.00)	NS		
57	Alanine (A)	3 (1.40)	21 (3.75)	NS		
	**Aspartic acid (D)**	**85 (39.72)**	**271 (48.39)**	**0.0359**	**0.7027**	**0.5102–0.9678**
	Serine (S)	15 (7.01)	63 (11.25)	NS		
	**Valine (V)**	**111 (51.87)**	**205 (36.61)**	**0.0002**	**1.866**	**1.357–2.566**
60	Histidine (H)	3 (1.40)	21 (3.75)	NS		
	**Serine (S)**	**111 (51.87)**	**205 (36.61)**	**0.0002**	**1.866**	**1.357–2.566**
	**Tyrosine (Y)**	**100 (46.73)**	**334 (59.64)**	**0.0016**	**0.5935**	**0.4322–0.8152**
78	**Valine (V)**	**76 (35.51)**	**131 (23.39)**	**0.0010**	**1.804**	**1.282–2.538**
	**Tyrosine (Y)**	**138 (64.49)**	**429 (76.61)**	**0.0010**	**0.5545**	**0.3940–0.7803**

OBI, occult hepatitis B infection; AA-F, amino acid frequency; OR, odds ratio; 95%CI, 95% confidence interval; *Pc*, corrected *P*. Gln: glutamine; Arg: Arginine; Un: Undetermined; Ala: alanine; Ser: Serine; Val: Valine; His: Histidine; Tyr: Tyrosine

In bold: significant value of *P* < 0.05

The amino acid residue on position 57 participates in the formation of a α-helix in the ninth peptide-binding pocket of HLA-DRβ1 chain [18]. Among the four possible amino acid residues, Alanine(A), Aspartic acid(D), Serine(S), Valine(V), at this position, Valine(V) showed a risk effect for OBI (*P* = 0.0002, OR = 1.866, 95% CI 1.357–2.566). Aspartic acid(D) showed a protective effect (*P* = 0.0359, OR = 0.7027, 95% CI 0.5102–0.9678), the other two residues had no significant effect.

A similar level of significance was observed for the amino acid residue on position 60 in the HLA-DRβ1 chain. This amino acid residue forms part of an α-helix in the ninth peptide-binding pocket of HLA-DRβ1 chain. Three possible amino acid residues, Histidine(H), Serine(S) and Tyrosine(Y), were identified at this position. Serine(S) (*P* = 0.0002, OR = 1.866, 95% CI 1.357–2.566) showed a risk effects for OBI. Tyrosine(Y) (*P* = 0.0016, OR = 0.5935, 95% CI 0.4322–0.8512) showed a protective effect. Histidine (H) had no significant association with OBI.

The amino acid residue on position 78 participates in the formation of a α-helix in the fourth peptide-binding pocket. Two possible amino acid residues, Valine(V) (*P* = 0.0010, OR = 1.804, 95% CI 1.282–2,538) showed risk effect for OBI. Tyrosine(Y) (*P* = 0.0010, OR = 0.5545, 95% CI 0.3940–0.7803) showed a protective effect.

### Analysis of genotype combination of HLA-DRB1 epitope

The analysis for homozygous and heterozygous genotype combination of HLA-DRB1 residues was presented (Table 6; Fig. 3b). The homozygous combinations in susceptible residues in OBI were: Val^57β^/Val^57β^, Ser^60β^/Ser^60β^. The heterozygous combinations in susceptible residues in OBI were: Gln^4β^/Arg^4β^, Asp^57β^/Val^57β^, Val^78β^/Tyr^78β^. The homozygous combinations in protectable residues in OBI were: Arg^4β^/Arg^4β^, Asp^57β^/Asp^57β^, Tyr^60β^/Tyr^60β^, Tyr^78β^/Tyr^78β^_._

**Table 6:** Homozygous and heterozygous genotype of HLA-DRB1 epitopes associated with OBI

Pocket	Genotype	OBI group (AA-GF%)	Control group (AA-GF%)	Disease association indices		
		(2n = 214)	(2n = 560)	*P* value	OR	95% CI
	Gln^4β^/Gln^4β^	10 (9.35)	13 (4.64)	0.080	2.117	0.899–4.986
	**Gln**^**4β**^**/Arg**^**4β**^	**56 (52.34)**	**105 (37.50)**	**0.008**	**1.830**	**1.167–2.870**
	**Arg**^**4β**^**/Arg**^**4β**^	**41 (38.32)**	**162 (57.86)**	**0.001**	**0.452**	**0.287–0.714**
P9	Ala^57β^/Asp^57β^	2 (1.87)	9 (3.21)	0.734	0.574	0.122–2.699
	Ala^57β^/Ser^57β^	0 (0.00)	5 (1.79)	0.328	1.018	1.002–1.034
	Ala^57β^/Val^57β^	1 (0.93)	7 (2.50)	0.453	0.368	0.045–3.026
	**Asp**^**57β**^**/Asp**^**57β**^	**10 (9.35)**	**58 (20.71)**	**0.009**	**0.395**	**0.194–0.804**
	Asp^57β^/Ser^57β^	7 (6.54)	32 (11.43)	0.153	0.543	0.232–1.269
	**Asp**^**57β**^**/Val**^**57β**^	**56 (52.34)**	**114 (40.71)**	**0.039**	**1.599**	**1.021–2.503**
	Ser^57β^/Ser^57β^	1 (0.93)	3 (1.07)	1.000	0.871	0.090–8.467
	Ser^57β^/Val^57β^	6 (5.61)	20 (7.14)	0.589	0.772	0.301–1.979
	**Val**^**57β**^**/Val**^**57β**^	**24 (22.43)**	**32 (11.43)**	**0.006**	**2.241**	**1.249–4.021**
P9	His^60β^/Ser^60β^	1 (0.93)	7 (2.50)	0.453	0.368	0.045–3.026
	His^60β^/Tyr^60β^	2 (1.87)	14 (5.00)	0.253	0.362	0.081–1.620
	**Ser**^**60β**^**/Ser**^**60β**^	**24 (22.43)**	**32 (11.43)**	**0.006**	**2.241**	**1.249–4.021**
	Ser^60β^/Tyr^60β^	61 (57.01)	134 (47.86)	0.107	1.445	0.922–2.263
	**Tyr**^**60β**^**/Tyr**^**60β**^	**19 (17.76)**	**93 (33.21)**	**0.003**	**0.434**	**0.249–0.756**
P4	Val^78β^/Val^78β^	10 (9.35)	13 (4.64)	0.080	2.117	0.899–4.986
	**Tyr**^**78β**^**/Tyr**^**78β**^	**42 (39.25)**	**162 (57.86)**	**0.001**	**0.471**	**0.299–0.742**
	**Val**^**78β**^**/Tyr**^**78β**^	**55 (51.40)**	**105 (37.50)**	**0.013**	**1.763**	**1.124–2.764**

OBI, occult hepatitis B infection; AA-GF, amino acid genotype frequency; OR, odds ratio; 95%CI, 95% confidence interval; *Pc*, corrected *P*. Gln: glutamine; Arg: Arginine; Un: Undetermined; Ala: alanine; Ser: Serine; Val: Valine; His: Histidine; Tyr: Tyrosine.

In bold: significant value of *P* < 0.05

### A specific amino acid motif of HLA-DRB1 mediates risk in OBI

The risk effects observed for Q^4β^-V^57β^-S^60β^-V^78^ can be attributed to association of these variants with allele of the HLA-DRB1*07:01, aligning with our initial observation of susceptible alleles was HLA-DRB1*07:01. Whereas the amino acid motif Arg^4β^-Asp^57β^/Ser^57β^-Tyr^60β^-Tyr^78β^which showed protectable could be attributed to association of HLA-DRB1*01:01, HLA-DRB1*08:03, HLA-DRB1*15:01 (these three alleles were not statistically different after Bonferroni correction).

## Discussion

The present study examined HLA-DRB1 alleles and amino acid residues in OBI carriers and control group in Shaanxi Han population. Extensive HLA polymorphism contributes to the selection of antigenic peptides for presentation to T lymphocytes resulting in different immune responses to infection among individual. The relationship between HLA DRB1alleles and outcome of HBV infection has been explored in some study. HLA-DRB1*13 is associated with natural convalescence from HBV infection among Korean, Gambian and American populations. While, some studies show that HLA-DRB1*07 is a risk factor for chronic HBV infection. Almarri et al. discovered an excess in HLA-DR7 in cases of chronic, present HBV infection [19]. Yang G et al. reported the HLA-DRB1*07 are markedly higher in the HBV-infected group in Han individuals in northwestern China [20]. Xu YY et al. reported the DRB1*07 is associated with the susceptibility of the infant to intrauterine HBV infection [21]. Hwang SH et al. and Cho SW et al. reported DR7 and haplotypes containing DR7 are associated with HBV chronicity among Koreans [22, 23]. In Thio CL, Wu YF and Almarri A’s study demonstrated that HLA-DR7 is a risk factor for chronic HBV infection [24, 25]. Mostly collaborative studies indicated that some of HLA genes have a protective or susceptive association with pathogenesis and development of HBV infection; however, lacking of evaluating the relationship of HLA-DRB1 alleles with OBI in Shaanxi Chinese Han population. While our finding agreed with numerous previous studies that the HLA-DRB1*07:01 allele is significantly associated with OBI. Five HLA-DRB1*07:01 restricted HBV helper T lymphocyte epitopes, FFLLTRILTIPQSLD [26], GMLPVCPLIPGSTTTNTG [27], TSLNFLGGSPVC [27], TTNTGPCKTCTTPAQG [27], WASVRFSWLSLLVPF [28] have been defined and identified in the Immune epitope database and analysis resource (IEDB) online server (http://www.iedb.org). The expression and type of HBV related epitopes in OBI have not been reported. A detailed understanding of HBV related epitope in OBI may potentially reveal the pathogenesis of OBI.

Determination of the structural of the DRB1 molecules associated with OBI could help in identifying the disease mechanism. Many studies reported that rheumatoid arthritis (RA) susceptibility is linked to amino acid residues within DRB1 peptide-binding groove. DRB1 alleles with a conserved sequence at 70–74 amino acid residues (the shared epitope) in the third hyper-variable region of DRB1 molecular are involved in RA etiology and pathogenesis [29–31]. Menconi et al. have identified DRβ-Tyr-26, DRβ-Leu-67, DRβ-Lys-71, and DRβ-Arg-74 are strongly associated with type 1A diabetes and autoimmune thyroid disease [32]. Shared amino acids in the peptide-binding pocket have been demonstrated in nasopharyngeal carcinoma [33], primary biliary cholangitis [34], Parkinson’s disease [35]. Sakai A et al. have reported that HLA-DRβ1 position 26 and HLA-DPβ1 position 84–87 are independently associated with anti-HBs production against HBV vaccine [36].

To decipher the precise structural elements mediating the HLA-DRB1 associations with OBI, we examined the contribution of individual amino acids of the HLA-DRB1 molecule to disease risk. The amino-acid signatures for the peptide-binding pockets of DRβ-chain reveal specific molecular change for predisposition and protection from the disease. The data demonstrate that susceptible associations related to residues Gln^4β^, Val^57β^ (P9), Ser^60β^ (P9) and Val^78β^(P4), whereas the amino acids Arg^4β^, Asp^57β^(P9), Tyr^60β^(P9) and Tyr^78β^(P4) showed protective associations.

The immune synapse (IS) is initiated by T cell receptor (TCR) interaction with peptide-major histocompatibility complex (pMHC) to form a specificity complex. MHC-peptide monomers bind but do not activate T cells. A dimer of MHC-peptide complexes is necessary and sufficient for initiation of T cell activation. HLA-DR crystals form homodimers of ab heterodimers, a structure that in vitro leads to improved sensitivity on the part of CD4^+^ T cells [16, 39]. The amino acid residue of DRB1 at position 4 is located on the outside of the antigen-binding groove. Arg^4β^ (a positively charged hydrophilic amino acid) is substituted for Gln^4β^ (common neutral amino acid). Gln^4β^ alleles might form an IS faster, precisely because of the location of this residue, near the amino-terminal domain of the β-chain, free to associate its neighboring chains, while not so easy to do in the case of Arg^4β^ molecules. In the next step research work, we will investigate the properties of HLA-DR7 as well as other β4Arg + alleles in their homozygous state (expression levels, molecular stability). The amino acid residues at position 57 and 60 influences the binding of antigen side chains associated with the P9 pocket. Previous studies have been suggested that the antigenic peptides bound to HLA-DR is influenced by interactions between pocket 6 and pocket 9[40]. β57 is within pocket 9, with Asp/non-Asp occupancy at this position determining the type of anchor residue at this pocket (mostly small aliphatic vs acidic) [16, 41]. The amino acid residue at 60 changes from Tyr to Ser, determining the type of anchor residue at this pocket (aromatic vs small) [42]. The amino acid residue at position 78 located in pocket 4 of the DR peptide-binding cleft [43], The P4 pocket of peptide binding groove can influence and/or modifying the peptide-binding repertoire of DR molecules. The exact mechanism by which Val^78β^ confers susceptibility to OBI is unknown. The change from the common neutral amino acid (Tyr) to hydrophobic amino acid (Val), the amino acid change possibility modifies the structure of the peptide binding cleft and altering the peptide binding properties.

Inferring polymorphic HLA-DRB1 amino acid positions from the alleles allows for interrogation of specific residues within the DRB1 binding pocket for association with OBI. We identified HLA-DRB1 amino acid positions 4, 57, 60 and 78 associated OBI, which have been associated with autoimmune disease: position 57 with Vogt-Kusanagi-Harada’s disease [44], positions 60 with multiple sclerosis [45, 46], position 78 with primary biliary cirrhosis [47], indicating the auto immunogenic potential of these HLA-DRB1 amino acid positions susceptible to OBI. The inheritance of two copies (homozygous genotype) and one copy (heterozygous genotype) of amino acid conferred a higher probability of risk and protect for OBI. The amino acids Arg^4β^, Asp^57β^, Tyr^60β^ and Tyr^78β^ show protective association with OBI, while the frequency of amino acid genotype Arg^4β^/Arg^4β^, Asp^57β^/Asp^57β^, Tyr^60β^/Tyr^60β^, Tyr^78β^/Tyr^78β^ in control group were higher than OBI carriers, also reveal protective. The frequency of heterozygous genotype Gln^4β^/Arg^4β^, Asp^57β^/Val^57β^, Val^78β^/Tyr^78β^ were higher in OBI carriers. Based on this result, we infer that certain types of amino acid affect the binding to antigenic peptides, resulting in the correlation between certain types of HLA-DRB1 and OBI. In doing so, we are able to identify the specific amino acid and elucidate structural features mediating both risk and protection in OBI, may the integrated influence of synergic effects, counteraction or offsetting effects.

The susceptibility effects observed for Gln^4β^, Val^57β^, Ser^60β^, Val^78β^ can be attributed to association of these variants with allele of HLA-DRB1*07:01, aligning with our initial observation of susceptibility mediated by DRB1*07:01. The protective effects observed for Arg^4β^, Asp^57β^/Ser^57β^, Tyr^60β^ and Tyr^78β^ can be attributed to association of these variants with allele of HLA-DRB1*01:01, HLA-DRB1*08:03, and HLA-DRB1*15:01, aligning with our initial observation of protective mediated by these alleles. All 4 residues of DRB1 are located either under (Arg/Gln^4β^ under the P9 pocket) or within the peptide-binding groove (β57, β60, β78), suggesting that specific patterns of these residues recognize specific peptides from viral antigens as the functional mechanism.

There are three primary limitations to this study. First, immune evasion caused by mutations has not yet been done in this study. Second, the 107 OBI samples were collected from 178,941 blood donors, 103 of them were serologically positive and 4 were serologically negative OBI. The differences in HLA-DRB1 alleles and amino acids between serologically positive and negative OBI have not been compared. And finally, the HBV infection patients have not been studied in this study. Next, our study group will carry out this part of research.

In summary, we find a clear role for HLA-DRB1 in predisposition and protection in OBI carriers. The association of specific combinations of amino acids that participate in critical peptide-binding pockets of the DRB1 molecule implies antigen presentation as a key factor in HBV and other viral infectious diseases.

## Conclusion

Our key finding are as follows: (1) HLA-DRB1*07:01 significantly associates with OBI, which agrees with several studies about chronic HBV persistence. (2) Determined and analyzed the amino acid sequence encoded by the DRB1 allele in relation to OBI. We can identify the specific amino acids and elucidate structural features mediating risk and protection to OBI. The amino acid variation at HLA-DRβ reveal susceptible associations for residues Gln^4β^, Val^57β^ (P9), Ser^60β^ (P9) and Val^78β^(P4), whereas the amino acids Arg^4β^, Asp^57β^(P9), Tyr^60β^(P9) and Tyr^78β^(P4) show protective associations.

## Data Availability

The data that support the finding of this study are available on request from the corresponding author. The data are not publicly available due to privacy or ethical restrictions.
